# Contrasting gut microbiota in captive Eurasian otters (*Lutra lutra*) by age

**DOI:** 10.1007/s00203-021-02526-w

**Published:** 2021-08-16

**Authors:** Yumiko Okamoto, Natsumi Ichinohe, Cheolwoon Woo, Sung-Yong Han, Hyeong-Hoo Kim, Sakura Ito, Chiho Nakamura, Junpei Kumura, Kentaro Nagaoka, Naomichi Yamamoto

**Affiliations:** 1 Hsinchu Zoo, Hsinchu, 300 Taiwan; 2 Laboratory of Veterinary Physiology, Department of Veterinary Medicine, Tokyo University of Agriculture and Technology, Tokyo, 183-0054 Japan; 3 Department of Environmental Health Science, Graduate School of Public Health, Seoul National University, Seoul, 08826 South Korea; 4 Korean Otter Research Center, Hwacheon, 24135 South Korea; 5 Yokohama Zoological Gardens ZOORASIA, Yokohama, Kanagawa 241-0001 Japan; 6 Aquamarine Fukushima, Marine Science Museum, Iwaki, Fukushima 971-8101 Japan; 7 Laboratory of Veterinary Anatomy, College of Veterinary Medicine, Seoul National University, Seoul, 08826 South Korea

**Keywords:** Ex situ conservation, Gastrointestinal tract, Microbiome, Microflora, Near threatened species, 16S rRNA gene

## Abstract

Understanding the gut microbiota characteristics of endangered species such as the Eurasian otter (*Lutra lutra*), especially in their early stages of life, could be essential for improving their management and ex situ conservation strategies. Here, we analyzed the gut microbiota diversity, composition, and function of captive Eurasian otters at different ages using high-throughput 16S rRNA gene sequencing. We found that: (1) Clostridiaceae was abundant in all age stages; (2) *Lactococcus* in cubs is thought to predominate for digesting milk; (3) bacteria associated with amino acid metabolism increase with age, while bacteria associated with carbohydrate metabolism decrease with age, which is likely due to decrease in dietary carbohydrate content (e.g., milk) and increase in dietary protein contents (e.g., fishes) with age; and (4) fish-related bacteria were detected in feces of healthy adults and juveniles. Overall, the gut microbiota of captive Eurasian otters was taxonomically and functionally different by age, which is thought to be attributed to the difference in the diet in their life stages. This study provided baseline information regarding the gut microbiota of Eurasian otters for the first time and contributes to improvement in their management in captivity.

**Supplementary Information:**

The online version contains supplementary material available at 10.1007/s00203-021-02526-w.

## Introduction

The Eurasian otter (*Lutra lutra*) belongs to the subfamily Lutrinae and is a top predator in freshwater ecosystems (Blanco-Garrido et al. 2008). Although the Eurasian otter was originally found throughout most of Europe, Asia, and North Africa, its population has declined in recent decades due to habitat destruction, pollution, and urbanization. Therefore, the Eurasian otter is currently designated as a near threatened species on the International Union for Conservation of Nature (IUCN) Red List and is listed in Appendix I of the Convention on International Trade in endangered species (CITES) to protect this species. In East Asia, it was declared in 2012 that the Eurasian otter has already become extinct in Japan (Nakanishi and Izawa 2019). The distribution area of the Eurasian otter has dramatically declined in China over the last half-century, and this species has not been recorded on Taiwan Island for over two decades (Li and Chan 2018). Moreover, the population of the Eurasian otter in Korea has also declined rapidly during the last several decades, even though it had once been abundant throughout the country (Jo and Won 2019).

Ex situ conservation (in captivity) is also very important in addition to in-situ conservation (in the wild) for the management of endangered species (Che-Castaldo et al. 2018). Among several approaches in ex situ conservation, studies on gut microbiota are essential because gut microbiota play an important role in maintaining the host’s health (Karmacharya et al. 2019). While it has been reported that hand-reared otter cubs have successfully grown up after being fed puppy milk replacers with low protein and lactose, several cubs have died as a result of weakness from diarrhea during the process (Ito et al. 2020). In humans, it is known that gut microbiota development influences infants’ health and subsequent host physiology (Moore and Townsend 2019). Therefore, gaining an understanding of the gut microbiota characteristics of the Eurasian otter in an early age may lead to improved husbandry skills of this endangered species. However, to the best of our knowledge, there is no valid information on the microbiota of the Eurasian otter during infancy, although several studies have been conducted using adult individuals (An et al. 2017; Finlayson-Trick et al. 2017) and other otter species, including the North American river otter (*Lontra canadensis*) (Finlayson-Trick et al. 2017; Guo et al. 2020a), neotropical otter (*Lontra longicaudis*) (Rodriguez-Rey and Santamaria-Vanegas 2020), and sea otter (*Enhydra lutris*) (Miller et al. 2010).

In this study, the gut microbiota diversity, composition, and function of Eurasian otters at different age stages were analyzed and compared using high-throughput 16S rRNA gene sequencing to provide baseline information regarding the gut microbiota of this species. As mentioned above, the Eurasian otter is extremely rare and ex situ conservation is essential. Research on physiology and ecology, including gut microbiota conditions, in captivity is vital for ex situ conservation, and this time, by clarifying the baseline characteristics of the gut microbiota of Eurasian otters, we aimed to improve husbandry management in captivity. To the best of our knowledge, this was the first observation of the gut microbiota characteristics of Eurasian otters in their early stages of life.

## Materials and methods

### Studied animals

A total of seven Eurasian otters kept at the Korean Otter Research Center were assessed in this study (Table 1; Fig. S1). Among them, three otters were born in captivity, and four otters were rescued in the wild and artificially raised. The age of rescued otter cubs was estimated based on their weight and length by a previously reported method (Reuther 1999). For comparison of the characteristics of gut microbiota by age, all individuals were categorized into four growth stages: cubs at the age of 1–2 M; cubs at the age of 3 M; cubs at the age of 4–5 M; and juveniles (8 M–2 Y) and adults (≥ 2 Y) (M = months, Y = years). The juvenile and adult otters were mainly fed 1 kg of Channel catfish (*Ictalurus punctatus*) once per day with seasonal feeding of landlocked salmon (*Oncorhynchus masou masou*) during winter. Conversely, the feeding of the cubs varied based on their developmental stage: (1) 50–80 ml of diluted Kitten Milk Replacer (KMR; Pet-Ag, Inc., Hampshire, IL, USA) was fed four times per day during 1–2 M; (2) 30 g of weaning food, including diluted Kitten Milk Replacer, defrosted Alaska pollock (*Gadus chalcogrammus*) (Frozen Alaska Pollack fillet; Youngwoo Corp., Busan, South Korea) and cans of kitten (Today’s recipe: Tuna pate, [Topper, Pocheon, South Korea] or Fancy Feast: Tender ocean whitefish feast kitten [Purina, Saint Louis, MO, USA]) or puppy food (Cesar Puppy beef and eggs, Mars Petcare, Wodonga, VIC, Australia), was fed four times per day during 3 M; and (3) 100–120 g of weaning food, including defrosted Alaska pollock and cans of kitten or puppy food, was fed three times per day during 4–5 M. No supplemental food was fed to any of the otters. Unfortunately, cub No. 5 died at 3 M, and cubs No. 6 and 7 died at 5 M due to unknown causes.

**Table 1:** Sample information

Otter ID	Growth stage	Sex	Sample name in NCBI	Sample ID	Estimated age at fecal sampling (Y, years; M, months, D, days)	Sampling date (YYMMDD)
1	Adult	Male	O1A	A1-1	2 Y (755 D)	190619
			O2A	A1-2	2 Y (760 D)	190624
3	Juvenile	Male	O3A	J1-1	8 M (241 D)	190619
			O4A	J1-2	8 M (246 D)	190624
4	Juvenile	Male	O5A	J2-1	1 Y (383 D)	190619
			O6A	J2-2	1 Y (388 D)	190624
2	Adult	Male	O7A	A2-1	2 Y (757 D)	190621
			O8A	A2-2	2 Y (760 D)	190624
5^a^	Cub	Male	O9Y	C1-2M-1	2 M (60 D)	180926
			O10Y	C1-2M-2	2 M (66 D)	181001
			O11Y	C1-2M-3	2 M (80 D)	181015
			O12Y	C1-3M-1	3 M (87 D)	181022
			O13Y	C1-3M-2	3 M (101 D)	181106
			O14Y	C1-3M-3	3 M (108 D)	181113
			O15Y	C1-4M-1	4 M (116 D)	181121
			O16Y	C1-4M-2	4 M (122 D)	181127
			O17Y	C1-4M-3	4 M (123 D)	181128
6^b^	Cub	Female	O18Y	C2-3M-1	3 M (90 D)	190110
			O19Y	C2-3M-2	3 M (103 D)	190123
			O20Y	C2-3M-3	3 M (110 D)	190130
			O21Y	C2-4M-1	4 M (118 D)	190207
			O22Y	C2-4M-2	4 M (132 D)	190221
			O23Y	C2-4M-3	4 M (144 D)	190304
			O24Y	C2-5M-1	5 M (153 D)	190313
			O25Y	C2-5M-2	5 M (158 D)	190318
7^c^	Cub	Female	O26Y	C3-1M-1	1 M (30 D)	181025
			O27Y	C3-1M-2	1 M (36 D)	181031
			O28Y	C3-1M-3	1 M (42 D)	181106
			O29Y	C3-1M-4	1 M (49 D)	181113
			O30Y	C3-2M-1	2 M (57 D)	181121
			O31Y	C3-2M-2	2 M (64 D)	181128
			O32Y	C3-2M-3	2 M (71 D)	181204
			O33Y	C3-3M-1	3 M (95 D)	181227
			O34Y	C3-3M-2	3 M (108 D)	190110
			O35Y	C3-4M-1	4 M (121 D)	190123
			O36Y	C3-4M-2	4 M (136 D)	190207
			O37Y	C3-5M-1	5 M (150 D)	190221
			O38Y	C3-5M-2	5 M (154 D)	190225
			O39Y	C3-5M-3	5 M (164 D)	190304
			O40Y	C3-5M-4	5 M (173 D)	190313

^a^Rescued at an estimated age of 2 M. Died at the age of 4 M (128 D)

^b^Rescued at an estimated age of 3 M. Died at the age of 5 M (161 D)

^c^Rescued at an estimated age of 1 M. Died at the age of 5 M (177 D)

### Fecal sample collection

In total, 40 fecal samples were collected noninvasively after natural voiding from September 2018 to June 2019. Fecal samples were collected immediately after excretion and stored frozen at − 20 °C until DNA extraction. The feces excreted on the dry floor for the marking purpose were collected to minimize contamination by water. All methods of sample collection were performed in accordance with a previously reported method (National Research Council 2011). Fecal samples were collected noninvasively by the animal keeper as part of normal rearing operations. Therefore, the approval by Institutional Animal Care and Use Committee was not required for this study.

### DNA extraction

The genomic DNA was extracted from about 0.5 g of each fecal sample by using the DNeasy PowerSoil Kit (QIAGEN, Venlo, The Netherlands) with modification (Hospodsky et al. 2010; An et al. 2017; Kumari et al. 2019). In addition to the DNeasy Powersoil Kit’s beads, 0.1-mm diameter glass beads (300 mg) and 0.5-mm diameter glass beads (100 mg) were supplemented for the 3-min physical disruption step by the mini beadbeater-24 (Biospec Products, Inc., Bartlesville, OK, USA). After bead beating, DNA was extracted, purified, and eluted to 50 μl of TE (10 mM Tris–HCl, 1 mM EDTA, pH = 8.0) based on the kit’s protocol. The extracted DNA samples were stored at − 80 °C until subsequent DNA sequencing analysis.

### DNA sequencing

The extracted DNA was amplified using universal bacterial primers targeting the V3 and V4 regions of the 16S rRNA gene (Herlemann et al. 2011) with MiSeq adapter sequences. The PCR was carried out in a 50 μl mixture composed of 2 × PCR Solution Premix Taq™ DNA polymerase (Takara Bio Inc., Otsu, Shiga, Japan), 1 μM of each primer, and 1 μl of each extract. The PCR conditions were as follows: 5 min at 95 °C for the denaturing step, followed by 35 cycles consisting of 30 s at 95 °C, 30 s at 55 °C, and 60 s at 72 °C. The final elongation step was performed for 10 min at 72 °C on a T100™ thermal cycler (Bio-Rad Laboratories, Inc., Hercules, CA, USA). The amplicons were purified using AMPure XP beads (Beckman Coulter, Inc., Brea, CA, USA) and indexed with a Nextera XT Index Kit v2 (Illumina, Inc., San Diego, CA, USA) in a 50 μl mixture consisting of 2 × PCR Solution Premix Taq™ DNA polymerase (Takara Bio Inc.), 5 μl of each index primer, and 5 μl of each purified DNA. The index PCR conditions were as follows: 3 min at 95 °C for the denaturing step, followed by 8 cycles consisting of 30 s at 95 °C, 30 s at 55 °C, and 30 s at 72 °C. The final elongation step was 5 min at 72 °C. The indexed PCR products were purified using AMPure XP beads (Beckman Coulter) and normalized to 4 nM with 10 mM Tris–HCl (pH = 8.5). The subsequent products were pooled with an internal control PhiX (30%) and loaded on a v3 600 cycle-kit reagent cartridge (Illumina, Inc.). The 2 × 300 bp paired-end sequencing was performed using an Illumina MiSeq (Illumina, Inc.). Raw sequence data are available in the Sequence Read Archive of the National Center for Biotechnology Information under project number PRJNA613985. The raw sequence reads were demultiplexed using MiSeq Reporter v2.5 (Illumina, Inc.) for subsequent bacterial taxonomical and functional analyses.

### Taxonomical analyses

The demultiplexed reads were processed using USEARCH version 11.0.667 (Edgar 2010). Forward and reverse reads were joined and low-quality reads with lengths less than 200 bp and/or > 1.0 expected errors were removed using the -fastq_filter command. Unique sequences were identified among joined and filtered reads using the -fastx_uniques command. Using the UNOISE3 algorithm (Edgar and Flyvbjerg 2015; Edgar 2016), the reads with sequencing errors and chimeric sequences were removed. Simultaneously, the resultant reads were clustered into zero-radius operational taxonomic units (ZOTUs) at a 100% sequence identity level (Edgar 2018b). Using the SINTAX algorithm (Edgar 2016, 2018a), each ZOTU was taxonomically assigned against the RDP training set v16 (rdp_16s_v16.fa.gz) with a cutoff value of 0.8. Additionally, α and β diversity analyses based on ZOTUs were performed by the “phyloseq” package (McMurdie and Holmes 2013) on R version 4.0.4. The Jaccard similarity coefficient and Bray–Curtis similarity coefficient were computed to analyze the similarity of bacterial assemblage memberships and structures across samples, respectively. Permutational multivariate analysis of variance (PERMANOVA) was performed to test the statistical difference in the gut microbiota across the age groups. The post hoc pairwise comparisons were performed by the “pairwiseAdonis” package (Martinez Arbizu 2020).

### Functional analyses

Phylogenetic Investigation of Communities by Reconstruction of Unobserved States (PICRUSt) (Langille et al. 2013) was used for functional analyses. The functional analyses were performed separately from the abovementioned taxonomic analyses from raw sequence reads according to the method of PICRUSt. Briefly, poly(N) tails of raw sequence reads were trimmed by the Trimmomatic-0.39 (Bolger et al. 2014). The forward and reverse reads were joined by the join_paired_ends.py script with a minimum allowed overlap of 10 bp (-j 10) on Quantitative Insights Into Microbial Ecology (Caporaso et al. 2010). The operational taxonomic units (OTUs) at a 97% sequence similarity threshold level were identified by the pick_closed_reference_otus.py script against the Greengenes database v13_5 (DeSantis et al. 2006; McDonald et al. 2012). The resultant OTUs were normalized by the normalize_by_copy_number.py script with the Greengenes database v13_5. The functions were predicted by the predict_metagenomes.py script with the Greengenes database v13_5 and Kyoto Encyclopedia of Genes and Genomes (KEGG) Orthologs (Kanehisa and Goto 2000; Kanehisa 2019; Kanehisa et al. 2021). Finally, the predicted functions were classified by the categorize_by_function.py script. The categorized functions were further analyzed by Statistical Analysis of Metagenomic Profiles version 2.1.3 (Parks et al. 2014) to compare the gut microbiota across the age groups.

## Results

### Sequencing statistics

After a series of quality filter steps, a total of 1,084,107 (average length of 448 bp) chimera-free high-quality sequences were recovered, with an average of 27,102 sequences per sample, ranging from 9749 to 45,373. These sequences were assigned to a total of 2548 ZOTUs at a 100% sequence identity level, which were sorted from 40 fecal samples. Each sample had 617 ± 259 ZOTUs on average, ranging from 234 to 1079.

### Diversity

**Fig. 1:**
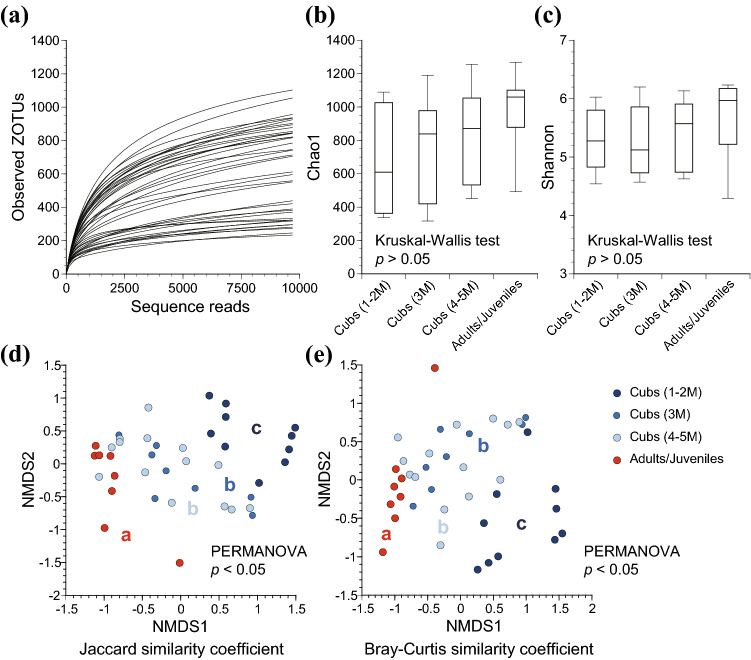
Diversity of gut microbiota of captive Eurasian otters across the age categories of 1–2 months, 3 months, 4–5 months, and adults/juveniles. Rarefaction curves based on zero-radius operational taxonomic units (ZOTUs) at a 100% sequence identity level (**a**). Alpha diversity metrics based on the Chao1 estimator (**b**) and Shannon index (**c**). The bottom and top lines of the boxes indicate the 25th and 75th percentiles, respectively, and the lines in the boxes indicates the medians. The bottom and top whiskers indicate the 10th and 90th percentiles, respectively. Beta diversity based on the Jaccard similarity coefficients (**d**) and Bray–Curtis similarity coefficient (**e**) on non-metric multidimensional scaling (NMDS) plots, with different letters (a, b, and c) representing significant differences (*p* < 0.05) by pair-wise comparisons

For diversity analyses, each library was rarefied to 9700 sequence reads. The rarefaction curves are shown in Fig. 1a. The α diversity analyses revealed increasing trends in the Chao1 estimator (species richness) (Fig. 1b) and the Shannon index (species diversity) (Fig. 1c) as the otters aged from birth to adulthood. However, the tendencies were not statistically significant (*p* > 0.05; Kruskal–Wallis test). The results of α diversity metrics for all samples are listed in Table S1. The β diversity analyses based on the Jaccard similarity coefficients (community membership) (Fig. 1d) and Bray–Curtis similarity coefficients (community structure) (Fig. 1e) revealed significant differences in the gut microbiota across the age categories (*p* < 0.05; PERMANOVA). The pairwise comparisons revealed differences in gut microbiota, both in terms of community structures and memberships, across the age categories, except between the groups of cubs at the ages of 3 M and 4–5 M.

### Taxonomic compositions

**Fig. 2:**
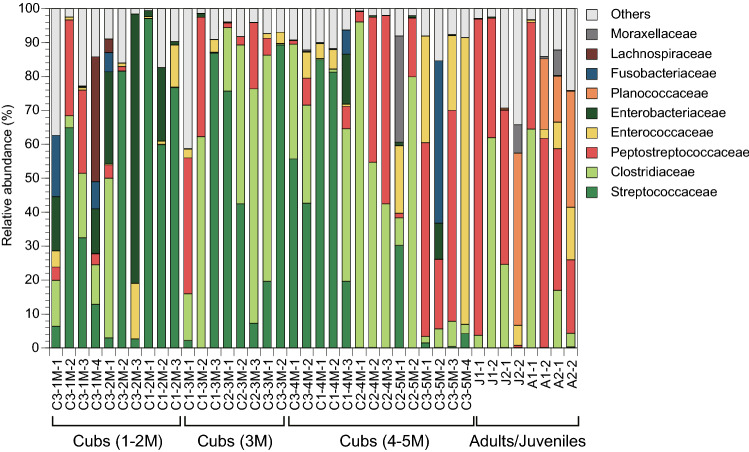
Relative abundance of bacterial families in gut microbiota of captive Eurasian otters. The alphabet character before the first hyphen in each sample ID represents “C” for cubs, “J” for juveniles, and “A” for adults. The number before the first hyphen indicates the otter's individual ID. For cubs, the number after the first hyphen represents the age in the month of the otter. The number after the last hyphen represents the sample ID within each sampling month for each otter

The relative abundances of gut microbiota at the family level are shown in Fig. 2. There was a tendency towards greater Peptostreptococcaceae abundance as the otters aged, while Streptococcaceae became less abundant with age. On average, relative abundances of Streptococcaceae were 44%, 40%, 23%, and 0.1% for cubs during 1–2 M, 3 M, and 4–5 M, and adults/juveniles, respectively. Conversely, Peptostreptococcaceae was more abundant in adults/juveniles than in cubs, i.e., on average 6.5%, 13%, 20%, and 41% for cubs during 1–2 M, 3 M, 4–5 M, and adults/juveniles, respectively. Furthermore, Enterobacteriaceae was particularly abundant in cubs during 1–2 M, i.e., on average 16%, while it was less abundant in other age groups, i.e., 0.2%, 2.0%, and 0.1% for cubs during 3 M and 4–5 M, and adults/juveniles, respectively. Clostridiaceae was abundant in all age groups, i.e., on average 9.5%, 35%, 29%, and 22% for cubs during 1–2 M, 3 M, and 4–5 M, and adults/juveniles, respectively.

**Fig. 3:**
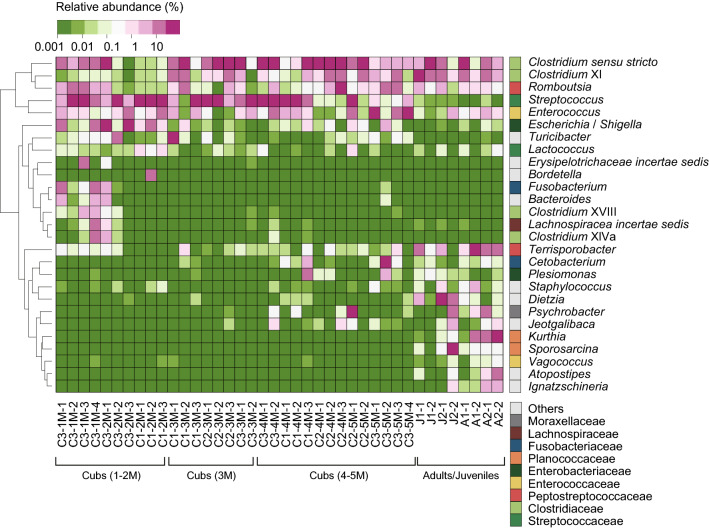
Relative abundance of identified bacterial genera or taxa in gut microbiota of captive Eurasian otters. The data of unidentified sequence reads are not included. The data shown represent 84.6% of all high-quality sequence reads. The alphabet character before the first hyphen in each sample ID represents “C” for cubs, “J” for juveniles, and “A” for adults. The number before the first hyphen indicates the otter’s individual ID. For cubs, the number after the first hyphen represents the age in the month of the otter. The number after the last hyphen represents the sample ID within each sampling month for each otter. The tree represents the Euclidean distance-based similarities of log-transformed bacterial compositions

The relative abundances of gut microbiota at the genus rank are shown in Fig. 3. In the family Streptococcaceae, *Streptococcus* and *Lactococcus* were identified, with mean relative abundances of 27% and 0.10%, respectively. They were the most abundant in cubs during 1–2 M. In the family Peptostreptococcaceae, *Terrisporobacter* was identified, with a mean relative abundance of 2.7% and the highest abundance observed in the group of adults and juveniles. In the family Enterobacteriaceae, *Escherichia*/*Shigella* and *Plesiomonas* were identified, with respective mean relative abundances of 4.2% and 0.51%. *Escherichia*/*Shigella* were the most abundant in cubs during 1–2 M, while *Plesiomonas* was abundant in cubs in 4–5 M and adults/juveniles. Four phylogenetic clusters of *Clostridium* were identified, i.e., *Clostridium *sensu stricto, *Clostridium* XI, *Clostridium* XlVa, and *Clostridium* XVIII with respective mean relative abundances of 23%, 5.6%, 0.67%, and 0.24%. *Clostridium *sensu stricto and *Clostridium* XI were detected in both cubs and adults/juveniles. However, *Clostridium* XlVa and *Clostridium* XVIII were detected only in cubs, with the highest relative abundances observed in the cub No. 2 during 1–2 M. The similar tendency was observed for *Bacteroides* and *Fusobacterium*. Conversely, some bacterial genera were more abundant in older cubs (4–5 M) and adults/juveniles. The examples include *Psychrobacter*, *Cetobacterium*, *Plesiomonas*, *Vagococcus*, *Dietzia*, and *Sporosarcina*.

### Functional tendencies

**Fig. 4:**
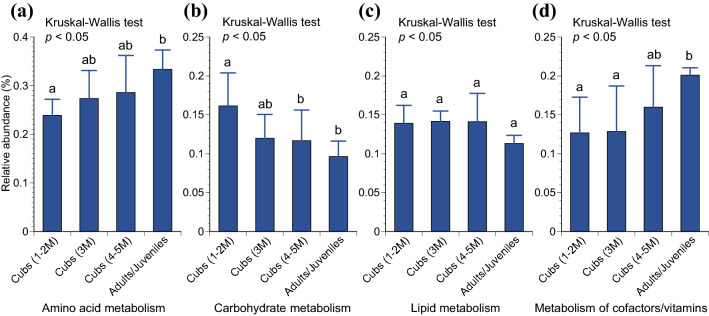
Relative abundance of genes associated with **a** amino acid metabolism, **b** carbohydrate metabolism, **c** lipid metabolism, and **d** metabolism of cofactors and vitamins in a metagenome predicted by Phylogenetic Investigation of Communities by Reconstruction of Unobserved States (PICRUSt). The results of significantly different predicted functions at the Kyoto Encyclopedia of Genes and Genomes (KEGG) level 2 functions are shown. The error bars represent standard deviations. The different letters (a, b, and c) representing significant differences (*p* < 0.05) by pair-wise comparisons based on the post hoc Tukey–Kramer test

PICRUSt predicted four of the KEGG level 2 functions that were significantly different across the age categories, i.e., amino acid metabolism, carbohydrate metabolism, lipid metabolism, and metabolism of cofactors and vitamins (*p* < 0.05; Kruskal–Wallis test) (Fig. 4). The relative abundance of genes associated with amino acid metabolism and metabolism of cofactors and vitamins were found to increase with age. Conversely, the relative abundance of genes associated with carbohydrate metabolism and lipid metabolism were found to decrease with age. Pairwise comparisons based on the post hoc Tukey–Kramer test confirmed statistical significance between the groups of cubs during 1–2 M and adults/juveniles (*p* < 0.05), except for lipid metabolism. All of the KEGG level 2 and 3 functions significantly different across the age categories are listed in Table S2.

## Discussion

Understanding gut microbiota is important for health management in captivity and ex situ conservation of endangered species because mammalian gut microbiota provides a range of essential functions for the host from the digestion of complex food to signaling the host immune system (McFall-Ngai et al. 2013). In this study, the first comparative investigation of the gut microbiota of captive Eurasian otters was conducted to characterize fecal microbial diversity, composition, and function with age.

Although it is not statistically significant, we found that the α diversity metrics of Eurasian otters increased with age (Fig. 1b, c), as previously reported in the forest musk deer (*Moschus berezovskii*) (Zhao et al. 2019a) and the Asian elephant (*Elephas maximus*) (Kambe et al. 2020), and that their gut microbiota structures became similar to those of adults as they aged. For carnivorous animals, previous studies of gut microbiota compared by gender, age, subspecies, or diet in the tiger (*Panthera tigris*) (Jiang et al. 2020) and spotted hyena (*Crocuta crocuta*) (Chen et al. 2020) showed that diet might be the most significant factor to shape the gut microbiota. In this study, significant differences in the β diversity and changes in the gut microbiota were observed in the four age groups with different dietary contents (Figs. 1d, e, 2, 3). The dietary contents are considered to be a factor that caused the observed differences.

Differences in relative abundance in gut microbiota were compared based on age stages, and Clostridiaceae was abundant in all age stages (Fig. 2), which was consistent with a study that revealed that the relative abundance of Clostridiaceae was 52% for Eurasian otters (An et al. 2017). Meanwhile, Enterococcaceae abundance appeared to be high in the gut microbiota of otters from 5 M to adults, which was supported by previous studies that showed that *Enterococcus* spp. belonging to the family Enterococcaceae were abundantly detected in wild Eurasian otters (Oliveira et al. 2008). The shift in the predominant family from Streptococcaceae to Peptostreptococcaceae with age was thought to have corresponded to their life stages. The bacterial genus *Streptococcus* belonging to the family Streptococcaceae is known to be abundantly detected in human newborn infants (Chong et al. 2018), and Peptostreptococcaceae is the representative bacterial family of carnivore species (Nishida and Ochman 2018).

At the genus rank, *Lactococcus* was dominant in the 1- to 2-month-old cubs, and *Terrisporobacter* was dominant in the adults and juveniles (Fig. 3). A study of the same Mustelidae American mink (*Neovison vison*) by Compo et al. (2018) showed that *Lactococcus* was abundantly detected in freshly weaned kits than adult females, which supports that the genus *Lactococcus* is thought to predominate for digesting milk. *Terrisporobacter* has been reported to be the most predominant in the gut microbiota of human infants fed only powder milk (Cai et al. 2019). In animals, *Terrisporobacter* was found in the gut microbiota of giant pandas (Zhao et al. 2019b) and pigs (McCormack et al. 2019). Notably, Wang et al. (2019) showed that pigs fed low-protein supplemented with casein hydrolysate (LPC) diet increased the abundance of *Lactobacillus* and decreased the abundance of *Terrisporobacter* compared with low-protein supplemented with free all amino acids (LPA) diet. Therefore, *Terrisporobacter* might be involved in the digestion of free all amino acids, but its roles in adult otters need further investigation.

Our functional analysis revealed that bacteria associated with amino acid metabolism and metabolism of cofactors and vitamins were found to increase with age, while bacteria associated with carbohydrate metabolism and lipid metabolism were found to decrease with age (Fig. 4). In the study for the tigers reported by Jiang et al. (2020), carbohydrate metabolism, amino acid metabolism, energy metabolism, and metabolism of cofactors and vitamins were reported as the main functions of their gut microbiota. Jiang et al. (2020) also reported the absence of *Candida*, a yeast, in the gut microbiota of meat-fed adult tigers and argued that it was attributable to a high protein content of the tiger diet. *Candida*, which is found in the gut microbiota of dogs and humans, is known to be related to digestion of carbohydrate-rich foods (Hoffmann et al. 2013). Additionally, Jiang et al. (2020) reported that energy metabolism was more abundant in meat-fed adult tigers than milk-fed tiger cubs, indicating that the gut of meat-fed tigers harbors more bacteria that assist energy metabolism. Milk contains large amounts of carbohydrates. For instance, lactose, a type of carbohydrates, is known to account for about 40% of the energy contained in human breast milk (Grenov et al. 2016). In the Eurasian otters investigated in this study, we expect that increase in amino acid metabolism and a decrease in carbohydrate metabolism with age was due to a decrease in dietary carbohydrate content (e.g., milk) and an increase in dietary protein contents (e.g., fishes) with age.

*Fusobacterium* and *Bacteroides*, which are possible human pathogens, were detected in the gut microbiota of the cub No. 1 at the age of 1 month (Fig. 3). *Fusobacterium* is known to be associated with diseases such as colorectal cancer and acute appendicitis in humans (Zhong et al. 2014; Brennan and Garrett 2016). However, *Fusobacteria* is known to be detected frequently and abundantly in the gut of predatory animals, such as seals (Nelson et al. 2013), dolphins (Nishida and Ochman 2018), cheetahs (Ley et al. 2008), leopards (Han et al. 2019), polar bears (Ley et al. 2008), blue foxes, silver foxes, raccoon dogs (Peng et al. 2018; Liu et al. 2020), fringe-lipped bats (Nishida and Ochman 2018), vultures (Roggenbuck et al. 2014), and alligators (Keenan et al. 2013). Moreover, Jiang et al. (2020) reported that abundances of *Fusobacterium* increased in meat-fed tigers compared with mix-fed and milk-fed tigers, implying that the high abundance of *Fusobacterium* is associated with the digestion of meat. Similarly, *Bacteroides* was reported to be dominant in the gut microbiota of adult and juvenile spottedd hyenas (Chen et al. 2020), Amur tigers (He et al. 2018), and dholes, implying that *Bacteroides* is known as protein and fat degraders (Arumugam et al. 2011). These findings indicated that *Fusobacterium* and *Bacteroides* are unlikely to act as serious pathogens in otter cubs, too.

Several bacterial genera were abundantly detected only in feces of adult and juvenile otters (Fig. 3). Of them, some genera are known to be associated with fishes, such as *Psychrobacter*, *Cetobacterium*, *Plesiomonas*, *Vagococcus*, *Dietzia*, and *Sporosarcina*. For instance, the first species of *Psychrobacter* was reported to be isolated from fishes (Juni and Heym 1986). *Cetobacterium* and *Plesiomonas* were identified from the gut of freshwater fishes (Sugita et al. 1993; Larsen et al. 2014). *Vagococcus* sp. was isolated from rainbow trout as a fish pathogen (Ruiz-Zarzuela et al. 2005). *Dietzia* was reported to be isolated from a drain of a fish-egg-processing plant (Yumoto et al. 2002), while *Sporosarcina* was reported to be isolated from minced fish meat (Tsuda et al. 2015). Moreover, *Sporosarcina* was isolated from seawater in Korea (Yoon et al. 2001). The detection of fish-related bacteria in healthy adults and juveniles indicates that they may participate as part of the normal gut microbiota of adult Eurasian otters.

Unfortunately, all of the three cubs died due to unknown reasons. These cubs were rescued in the wild and artificially raised at the Korean Otter Research Center. Although the cause of cubs’ mortality is unknown, no particular abnormalities were observed in the health condition until shortly before death. In addition, there was no distinct difference between samples collected immediately before death in each individual (C1-4M-3, C2-5M-2, and C3-5M-4) and the samples collected before that. However, it is difficult to assert the relationship between changes in the gut microbiota and mortality because there were no surviving cubs to compare with the cubs that died at this time. We look forward to further analysis using surviving individuals as controls in the future. Meanwhile, in the first reported case of successful hand-rearing of a Eurasian otter cub abandoned by the parent otters in a captive environment in Japan, probiotic bacteria, such as probiotic strains of *Enterococcus faecium* and *Lactobacillus acidophilus*, were administered from 33 days-old (Ito et al. 2020). We do not know how probiotic bacteria if administered, could have improved the survivability of our rescued Eurasian otter cubs. Our study revealed that *Lactococcus* was dominant in the gut microbiota of 1- to 2-month-old Eurasian otter cubs, rather than *Lactobacillus*, which is reported to be predominant in giant panda weaning cubs (Guo et al. 2020b). Further research is warranted to investigate the roles of probiotic bacteria, including lactic acid bacteria such as *Lactococcus* and *Lactobacillu*s, in the health status of Eurasian otters in their early stages of life.

As a part of ex situ conservation activity, understanding the characteristics of the gut microbiota of endangered species in captivity is important. However, it should be noted that animals in captivity experience a range of changes that may influence the gut bacteria, such as diet changes, treatments, and reduced contact with other individuals, species, and variable environmental substrates that act as sources of bacterial diversity (McKenzie et al. 2017). These effects have been confirmed in many animals, such as nonhuman primates (Clayton et al. 2016, 2018), carnivores (Chong et al. 2019; Guo et al. 2019), and herbivores (Gibson et al. 2019; Zhao et al. 2019a). In otters, there is only one report in the neotropical otter (*Lontra longicaudis*) (Rodriguez-Rey and Santamaria-Vanegas 2020); therefore, further research is necessary to reveal the captive effects on the gut microbiota of the Eurasian otter and on the resultant possible health consequences.

It should also be noted that seasons can have a certain impact on the gut microbiota of animals. For instance, the significant seasonal differences in gut microbiota were reported by studies in giant pandas (Guo et al. 2020b) and Canadian mink of the same Mustelidae (Compo et al. 2018). In our study, we found the statistically significant differences in the gut microbiota collected between summer and fall, and between summer and winter (*p* < 0.05; PERMANOVA) (Table S3). We expect that the difference is due to the fact that all of juvenile/adult fecal samples were collected in the summer. The season effects appear to be much smaller than the age effects since the seasonal differences were observed only for summer. Moreover, if we look only at the results of cubs, significant differences were observed by age (Fig. 1d, e), but not by season (Table S3). Specifically, we observed statistically significant differences between 1–2 and 3–5 M (Fig. 1d, e), but no significant seasonal differences between the autumn, winter, and spring in which all of cubs’ feces were collected (Table S3). Furthermore, we observed the age-sequential increases in the α diversity measures, i.e., Chao1 estimator and Shannon index, although it was not statistically significant (Fig. 1b, c). Similarly, our functional analysis revealed that relative abundances of genes related to each metabolism increase or decrease sequentially by age (Fig. 4). These results imply that the effect of age was greater than the effect of the season since these age-sequential tendencies may have been more obscured if other confounding factors such as seasons predominated. However, future research should completely eliminate confounding factors such as season for better discerning the age effect.

In conclusion, we provided the first report of the dominant bacteria in the gut microbiota of Eurasian otters by age. The distinct differences in the gut microbiota and functions by age were observed, which is thought to be attributed to the difference in the diet in their life stages. However, the roles of these bacteria in otter’s health and diseases remain unknown. Characterization of bacteria that increase the survivability of the rescued cubs, which may include known human probiotics as well as fish-related bacteria commonly found in healthy adult otters, is necessary. This study investigated gut microbiota of Eurasian otters by age for the first time. We expect that the accumulation of knowledge of their gut microbiota will help elucidating the beneficial microbes. Studies on gut microbiota may be of particular relevance to ex situ conservation of wildlife in captive environments, such as zoos, which require preventative health care as well as veterinary treatment to increase the survivability of animals rescued in the wild or abandoned by the parents.

## Supplementary Information

Below is the link to the electronic supplementary material.

Supplementary file1 (PDF 838 kb)

## Data Availability

Raw sequence data are available in the Sequence Read Archive of the National Center for Biotechnology Information under project number PRJNA613985.
