# Supplementary material for: Contrasting gut microbiota in captive Eurasian otters (Lutra lutra) by age
Source: Arch Microbiol. 2021 Aug 16;203(9):5405–16. doi: 10.1007/s00203-021-02526-w (PMC8502154; doi:10.1007/s00203-021-02526-w)
Supplement: Supplementary file 1 — Supplementary file1 (PDF 838 kb) [file 203_2021_2526_MOESM1_ESM.pdf]

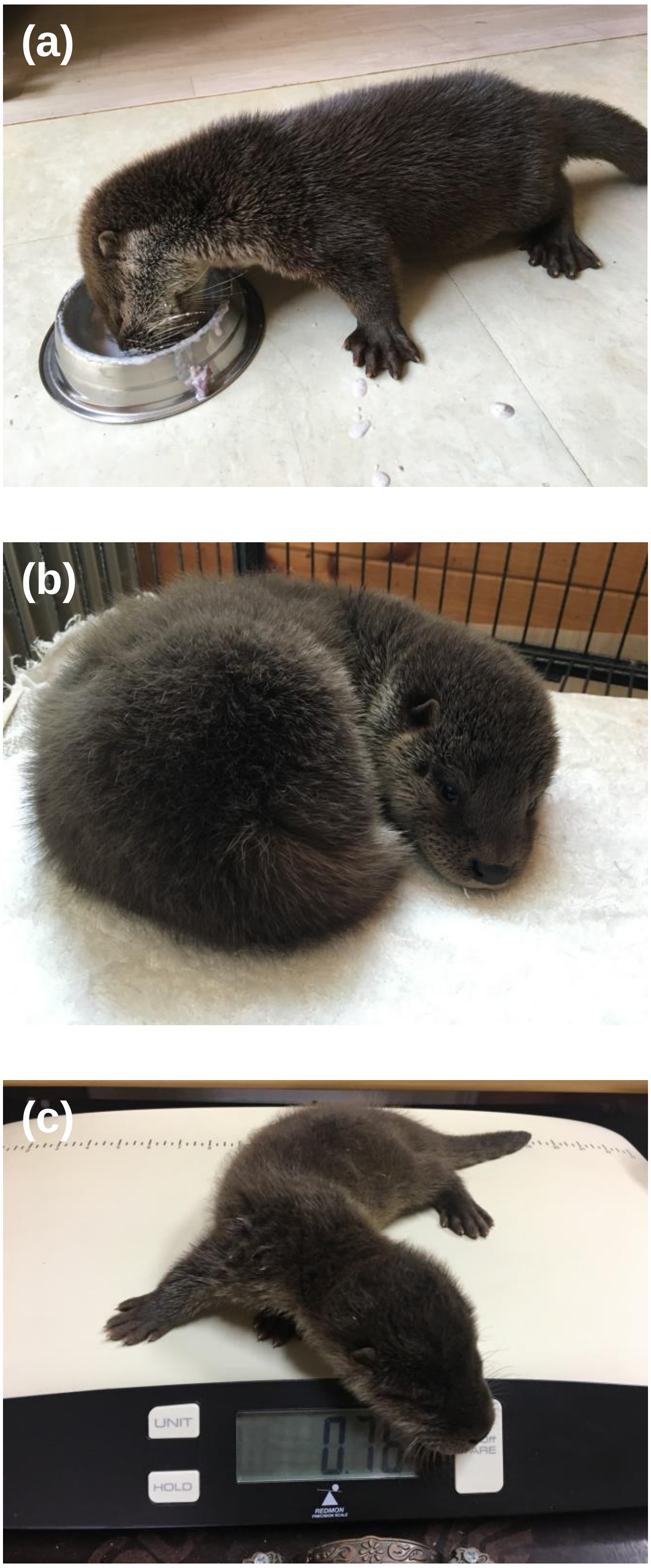

Figure S1. Rescued Eurasian otter cubs no. 5 (**a**), no. 6 (**b**), and no. 7 (**c**) artificially raised at the Korean Otter Research Center.

Table S1. Alpha diversity metrics based on zero-radius operational taxonomic units (ZOTUs) obtained from 9,700 rarified sequence reads.

Sample ID	Observed ZOTUs	Chao1	ACE	Shannon	Simpson	Inverse Simpson	Fisher
A1-1	933	1111	1130	5.92	0.995	193	254
A1-2	552	766	783	5.18	0.990	98	127
J1-1	878	1068	1013	6.25	0.997	385	234
J1-2	821	990	950	6.02	0.996	258	214
J2-1	937	1090	1039	6.20	0.997	286	256
J2-2	296	375	389	3.91	0.961	26	58
A2-1	1055	1334	1295	6.15	0.995	218	301
A2-2	785	1051	1051	5.26	0.977	43	202
C1-2M-1	246	325	336	4.35	0.983	57	46
C1-2M-2	333	386	386	5.17	0.992	124	67
C1-2M-3	274	363	340	4.73	0.987	79	52
C1-3M-1	846	969	955	5.95	0.996	228	223
C1-3M-2	1103	1276	1262	6.30	0.996	237	320
C1-3M-3	233	286	290	4.56	0.986	72	43
C1-4M-1	296	453	444	4.64	0.986	74	58
C1-4M-2	318	434	447	4.73	0.987	80	63
C1-4M-3	925	1256	1273	5.91	0.994	161	251
C2-3M-1	439	736	814	4.87	0.988	85	95
C2-3M-2	382	450	464	4.97	0.987	76	79
C2-3M-3	821	941	919	5.77	0.994	163	214
C2-4M-1	421	532	527	4.74	0.986	73	90
C2-4M-2	903	1054	1028	6.18	0.997	311	243
C2-4M-3	746	875	842	6.05	0.997	304	188
C2-5M-1	612	867	857	5.71	0.995	204	145
C2-5M-2	742	946	949	5.43	0.992	120	187
C3-1M-1	895	1083	1062	6.16	0.997	321	240
C3-1M-2	559	722	695	5.50	0.991	108	129
C3-1M-3	714	977	977	5.38	0.983	58	178
C3-1M-4	855	1026	964	5.89	0.993	150	226
C3-2M-1	846	1095	1059	5.80	0.995	198	223
C3-2M-2	371	496	535	4.83	0.989	88	76
C3-2M-3	323	348	341	5.05	0.990	105	64
C3-3M-1	706	987	953	5.27	0.989	94	175
C3-3M-2	281	388	391	4.59	0.986	71	54
C3-4M-1	390	594	657	4.94	0.986	70	81
C3-4M-2	843	1042	1044	5.83	0.994	174	222
C3-5M-1	596	813	838	5.34	0.993	137	140
C3-5M-2	817	1072	1044	5.75	0.994	165	213
C3-5M-3	957	1258	1235	6.13	0.997	316	263
C3-5M-4	323	467	451	4.52	0.984	64	64

Table S2. Relative abundance of predicted genes related to the Kyoto Encyclopedia of Genes and Genomes (KEGG) functions at levels 2 and 3. Only significantly different functions across the age categories are listed.

Function	A/J (%)	C(4–5M) (%)	C(3M) (%)	C(1–2M) (%)	Corrected *p* value [a]	A/J vs. C(4–5M)[b]	A/J vs. C(3M)[b]	A/J vs. C(1–2M)[b]	C(4–5M) vs. C(3M)[b]	C(4–5M) vs. C(1–2M)[b]	C(3M) vs. C(1–2M)[b]
Clavulanic acid biosynthesis	5.97E-05	0.00E+00	0.00E+00	0.00E+00	3.74E-07	****	****	****	n.s.	n.s.	n.s.
Biosynthesis of type II polyketide products	6.22E-04	9.00E-07	0.00E+00	1.32E-05	7.90E-06	*	*	*	n.s.	n.s.	n.s.
Synthesis and degradation of ketone bodies	1.47E-01	8.89E-02	7.42E-02	5.23E-02	2.60E-05	****	****	****	n.s.	*	n.s.
Ethylbenzene degradation	9.88E-02	7.70E-02	7.23E-02	4.86E-02	3.41E-05	**	***	****	n.s.	****	***
Hematopoietic cell lineage	1.96E-03	1.24E-05	8.92E-06	5.47E-07	6.76E-05	**	*	*	n.s.	n.s.	n.s.
Benzoate degradation	4.86E-01	3.37E-01	3.31E-01	2.79E-01	0.000123	***	***	****	n.s.	n.s.	n.s.
Insulin signaling pathway	4.32E-02	5.23E-02	4.93E-02	6.55E-02	0.000178	n.s.	n.s.	****	n.s.	**	**
mRNA surveillance pathway	2.82E-04	1.65E-06	5.60E-06	2.85E-06	0.000186	*	n.s.	*	n.s.	n.s.	n.s.
Circadian rhythm - plant	2.60E-03	2.14E-05	1.26E-05	2.90E-05	0.000199	n.s.	n.s.	n.s.	n.s.	n.s.	n.s.
Styrene degradation	4.89E-02	3.01E-02	2.03E-02	1.86E-02	0.000258	*	***	****	n.s.	n.s.	n.s.
Renin-angiotensin system	2.22E-03	1.42E-05	1.17E-05	3.38E-05	0.000282	**	*	*	n.s.	n.s.	n.s.
Parkinson's disease	4.48E-03	5.00E-03	1.06E-05	4.01E-04	0.000337	n.s.	n.s.	n.s.	n.s.	n.s.	n.s.
PPAR signaling pathway	1.31E-01	6.37E-02	4.41E-02	4.39E-02	0.000352	***	****	****	n.s.	n.s.	n.s.
Type II diabetes mellitus	3.96E-02	4.22E-02	4.82E-02	5.04E-02	0.000409	n.s.	**	****	n.s.	***	n.s.
Steroid biosynthesis	7.87E-03	1.03E-03	7.91E-05	2.28E-05	0.000414	**	**	***	n.s.	n.s.	n.s.
Indole alkaloid biosynthesis	3.24E-04	4.27E-06	1.93E-06	3.35E-06	0.000414	*	n.s.	*	n.s.	n.s.	n.s.
Caffeine metabolism	1.96E-03	2.39E-05	1.64E-05	1.01E-05	0.000415	**	*	*	n.s.	n.s.	n.s.
Meiosis - yeast	8.09E-03	1.62E-04	1.10E-04	1.75E-04	0.000495	***	**	**	n.s.	n.s.	n.s.
Butanoate metabolism	1.01E+00	8.43E-01	7.83E-01	7.34E-01	0.000522	***	****	****	n.s.	n.s.	n.s.
Cardiac muscle contraction	3.27E-03	4.01E-03	5.60E-06	3.51E-04	0.000552	n.s.	n.s.	n.s.	n.s.	n.s.	n.s.
Amoebiasis	4.45E-03	6.04E-04	3.61E-04	1.46E-04	0.000556	**	*	**	n.s.	n.s.	n.s.
Atrazine degradation	4.83E-02	2.62E-02	1.84E-02	1.11E-02	0.000584	*	***	****	n.s.	n.s.	n.s.
Vasopressin-regulated water reabsorption	1.11E-05	0.00E+00	0.00E+00	0.00E+00	0.000621	***	**	***	n.s.	n.s.	n.s.
Cytochrome P450	2.05E-03	1.69E-05	2.01E-05	5.38E-06	0.000664	**	*	*	n.s.	n.s.	n.s.
Fluorobenzoate degradation	2.18E-02	3.82E-04	1.66E-04	4.38E-03	0.000708	**	*	n.s.	n.s.	n.s.	n.s.
Betalain biosynthesis	3.80E-05	7.52E-07	0.00E+00	3.90E-06	0.000780	****	****	***	n.s.	n.s.	n.s.
Pathogenic Escherichia coli infection	0.00E+00	0.00E+00	0.00E+00	6.02E-05	0.000846	n.s.	n.s.	*	n.s.	***	*
Glycosaminoglycan degradation	4.64E-03	2.05E-02	2.85E-02	3.29E-02	0.000942	n.s.	***	****	n.s.	n.s.	n.s.
Naphthalene degradation	2.75E-01	2.09E-01	1.71E-01	1.55E-01	0.000954	n.s.	n.s.	n.s.	n.s.	n.s.	n.s.
Phenylalanine metabolism	2.35E-01	1.51E-01	1.24E-01	1.40E-01	0.00110	***	***	***	n.s.	n.s.	n.s.
Chaperones and folding catalysts	7.77E-01	8.04E-01	8.14E-01	8.93E-01	0.00114	n.s.	n.s.	***	n.s.	***	*
Colorectal cancer	1.21E-03	9.89E-04	4.98E-06	4.98E-05	0.00116	n.s.	n.s.	n.s.	n.s.	n.s.	n.s.
Influenza A	1.21E-03	9.89E-04	4.98E-06	4.98E-05	0.00116	n.s.	n.s.	n.s.	n.s.	n.s.	n.s.
p53 signaling pathway	1.26E-03	9.89E-04	4.98E-06	4.98E-05	0.00116	n.s.	n.s.	n.s.	n.s.	n.s.	n.s.
Small cell lung cancer	1.21E-03	9.89E-04	4.98E-06	4.98E-05	0.00116	n.s.	n.s.	n.s.	n.s.	n.s.	n.s.
Toxoplasmosis	1.21E-03	9.89E-04	4.98E-06	4.98E-05	0.00116	n.s.	n.s.	n.s.	n.s.	n.s.	n.s.
Viral myocarditis	1.21E-03	9.89E-04	4.98E-06	4.98E-05	0.00116	n.s.	n.s.	n.s.	n.s.	n.s.	n.s.
Protein digestion and absorption	2.12E-03	6.96E-05	1.03E-05	1.09E-03	0.00119	*	n.s.	n.s.	n.s.	n.s.	n.s.
Valine, leucine and isoleucine degradation	5.20E-01	2.83E-01	2.43E-01	2.40E-01	0.00122	***	***	***	n.s.	n.s.	n.s.

Ribosome Biogenesis	1.21E+00	1.35E+00	1.46E+00	1.46E+00	0.00131	n.s.	***	***	n.s.	n.s.	n.s.
Bacterial invasion of epithelial cells	2.06E-04	2.33E-04	4.72E-05	5.00E-03	0.00134	n.s.	n.s.	n.s.	n.s.	*	n.s.
Lipopolysaccharide biosynthesis proteins	6.77E-02	1.37E-01	1.18E-01	3.42E-01	0.00140	n.s.	n.s.	***	n.s.	***	**
Amyotrophic lateral sclerosis (ALS)	2.64E-02	5.98E-03	1.98E-03	1.63E-02	0.00144	n.s.	n.s.	n.s.	n.s.	n.s.	n.s.
Bile secretion	1.11E-04	3.40E-04	4.20E-06	3.00E-05	0.00190	n.s.	n.s.	n.s.	n.s.	n.s.	n.s.
Fatty acid metabolism	5.09E-01	2.91E-01	2.31E-01	2.54E-01	0.00193	***	***	***	n.s.	n.s.	n.s.
Bacterial secretion system	4.59E-01	4.81E-01	4.89E-01	6.21E-01	0.00199	n.s.	n.s.	***	n.s.	***	**
Renal cell carcinoma	8.26E-03	1.31E-03	1.67E-03	6.81E-03	0.00206	n.s.	n.s.	n.s.	n.s.	n.s.	n.s.
Ubiquitin system	1.10E-02	1.74E-03	1.52E-04	7.11E-03	0.00211	n.s.	*	n.s.	n.s.	n.s.	n.s.
Membrane and intracellular structural molecules	2.66E-01	3.34E-01	3.44E-01	5.12E-01	0.00213	n.s.	n.s.	****	n.s.	***	*
Endocytosis	8.67E-04	9.96E-04	0.00E+00	4.30E-06	0.00249	n.s.	n.s.	n.s.	n.s.	n.s.	n.s.
Fc gamma R-mediated phagocytosis	8.67E-04	9.96E-04	0.00E+00	4.30E-06	0.00249	n.s.	n.s.	n.s.	n.s.	n.s.	n.s.
GnRH signaling pathway	8.67E-04	9.96E-04	0.00E+00	4.30E-06	0.00249	n.s.	n.s.	n.s.	n.s.	n.s.	n.s.
Basal transcription factors	4.74E-03	9.44E-04	4.81E-04	1.49E-03	0.00255	n.s.	n.s.	n.s.	n.s.	n.s.	n.s.
Chagas disease (American trypanosomiasis)	4.43E-03	5.78E-04	8.26E-05	5.68E-03	0.00269	n.s.	n.s.	n.s.	n.s.	n.s.	n.s.
Prion diseases	6.06E-03	1.85E-03	1.81E-03	7.48E-03	0.00291	n.s.	n.s.	n.s.	n.s.	n.s.	n.s.
Systemic lupus erythematosus	9.53E-06	4.76E-07	0.00E+00	2.34E-06	0.00309	***	**	n.s.	n.s.	n.s.	n.s.
Bladder cancer	2.64E-03	5.63E-04	7.98E-05	5.56E-03	0.00348	n.s.	n.s.	n.s.	n.s.	*	*
Limonene and pinene degradation	2.44E-01	1.32E-01	1.09E-01	1.14E-01	0.00430	*	**	**	n.s.	n.s.	n.s.
Purine metabolism	2.01E+00	2.19E+00	2.32E+00	2.30E+00	0.00451	n.s.	*	*	n.s.	n.s.	n.s.
Lysine degradation	2.94E-01	1.51E-01	1.22E-01	1.41E-01	0.00461	**	***	**	n.s.	n.s.	n.s.
Pertussis	2.92E-03	4.75E-03	4.45E-04	6.44E-02	0.00462	n.s.	n.s.	**	n.s.	***	**
Tropane, piperidine and pyridine alkaloid biosynthesis	1.73E-01	1.28E-01	1.19E-01	9.31E-02	0.00466	n.s.	n.s.	***	n.s.	n.s.	n.s.
Tryptophan metabolism	3.30E-01	1.66E-01	1.33E-01	1.75E-01	0.00469	**	***	*	n.s.	n.s.	n.s.
DNA replication	5.80E-01	6.35E-01	6.99E-01	6.64E-01	0.00476	n.s.	***	*	n.s.	n.s.	n.s.
Propanoate metabolism	7.39E-01	5.71E-01	5.62E-01	5.86E-01	0.00487	**	**	*	n.s.	n.s.	n.s.
Chromosome	1.32E+00	1.42E+00	1.50E+00	1.48E+00	0.00489	n.s.	***	***	n.s.	n.s.	n.s.
Arginine and proline metabolism	1.30E+00	1.19E+00	1.14E+00	1.01E+00	0.00490	n.s.	n.s.	***	n.s.	*	n.s.
Carbon fixation pathways in prokaryotes	1.03E+00	9.33E-01	8.93E-01	8.68E-01	0.00498	n.s.	n.s.	*	n.s.	n.s.	n.s.
Flavonoid biosynthesis	3.43E-03	1.33E-03	1.25E-04	1.46E-03	0.00501	n.s.	n.s.	n.s.	n.s.	n.s.	n.s.
Homologous recombination	7.99E-01	8.71E-01	9.56E-01	9.02E-01	0.00501	n.s.	***	n.s.	n.s.	n.s.	n.s.
Glycosphingolipid biosynthesis - ganglio series	2.57E-03	6.16E-03	1.51E-03	1.04E-02	0.00501	n.s.	n.s.	n.s.	n.s.	n.s.	*
Chlorocyclohexane and chlorobenzene degradation	4.21E-02	1.04E-02	1.37E-03	7.58E-03	0.00512	n.s.	*	*	n.s.	n.s.	n.s.
Biosynthesis of type II polyketide backbone	2.30E-05	0.00E+00	0.00E+00	0.00E+00	0.00552	*	n.s.	n.s.	n.s.	n.s.	n.s.
Non-homologous end-joining	1.50E-02	5.44E-04	3.32E-03	2.51E-03	0.00627	*	n.s.	n.s.	n.s.	n.s.	n.s.
Glycosphingolipid biosynthesis - globo series	2.66E-02	6.24E-02	6.64E-02	7.58E-02	0.00629	*	n.s.	***	n.s.	n.s.	n.s.
Metabolism of xenobiotics by cytochrome P450	5.28E-02	2.96E-02	8.84E-03	3.47E-02	0.00681	n.s.	*	n.s.	n.s.	n.s.	n.s.
Cell cycle - Caulobacter	4.02E-01	4.36E-01	4.74E-01	4.56E-01	0.00684	n.s.	**	n.s.	n.s.	n.s.	n.s.
Biosynthesis of siderophore group nonribosomal peptides	2.93E-02	1.81E-02	6.64E-03	3.52E-02	0.00688	n.s.	n.s.	n.s.	n.s.	n.s.	*
DNA repair and recombination proteins	2.56E+00	2.70E+00	2.90E+00	2.79E+00	0.00703	n.s.	***	n.s.	n.s.	n.s.	n.s.
Metabolism of cofactors and vitamins	2.01E-01	1.60E-01	1.28E-01	1.27E-01	0.00718	n.s.	*	**	n.s.	n.s.	n.s.
N-Glycan biosynthesis	5.77E-03	1.65E-03	2.00E-04	3.02E-03	0.00719	n.s.	n.s.	n.s.	n.s.	n.s.	n.s.
beta-Alanine metabolism	2.89E-01	1.46E-01	1.14E-01	1.51E-01	0.00740	***	***	**	n.s.	n.s.	n.s.

Stilbenoid, diarylheptanoid and gingerol biosynthesis	1.30E-02	2.01E-03	6.13E-04	1.38E-03	0.00749	*	*	*	n.s.	n.s.	n.s.
DNA replication proteins	1.05E+00	1.18E+00	1.32E+00	1.22E+00	0.00774	n.s.	***	n.s.	n.s.	n.s.	n.s.
Translation factors	4.30E-01	4.78E-01	5.24E-01	4.99E-01	0.00776	n.s.	***	*	n.s.	n.s.	n.s.
Retinol metabolism	4.59E-02	2.59E-02	8.73E-03	2.15E-02	0.00829	n.s.	**	n.s.	n.s.	n.s.	n.s.
Drug metabolism - cytochrome P450	5.83E-02	3.05E-02	1.08E-02	3.52E-02	0.00895	n.s.	*	n.s.	n.s.	n.s.	n.s.
Flavone and flavonol biosynthesis	8.41E-04	1.19E-02	2.12E-02	1.77E-02	0.00924	n.s.	n.s.	n.s.	n.s.	n.s.	n.s.
Geraniol degradation	1.52E-01	4.44E-02	2.74E-02	6.92E-02	0.00973	n.s.	n.s.	n.s.	n.s.	n.s.	n.s.
Ribosome	2.01E+00	2.23E+00	2.49E+00	2.26E+00	0.0100	n.s.	**	n.s.	n.s.	n.s.	n.s.
Carbohydrate metabolism	9.64E-02	1.17E-01	1.20E-01	1.61E-01	0.0102	n.s.	n.s.	***	n.s.	*	n.s.
Ubiquinone and other terpenoid-quinone biosynthesis	1.28E-01	9.31E-02	2.89E-02	1.50E-01	0.0103	n.s.	n.s.	n.s.	n.s.	n.s.	n.s.
Taurine and hypotaurine metabolism	8.77E-02	9.71E-02	1.00E-01	1.10E-01	0.0105	n.s.	n.s.	***	n.s.	n.s.	n.s.
Lipid metabolism	1.13E-01	1.41E-01	1.42E-01	1.39E-01	0.0106	n.s.	n.s.	n.s.	n.s.	n.s.	n.s.
Proximal tubule bicarbonate reclamation	4.04E-02	2.39E-02	2.22E-02	1.56E-02	0.0108	n.s.	n.s.	***	n.s.	n.s.	n.s.
Lysosome	1.60E-02	2.81E-02	3.22E-02	3.81E-02	0.0109	n.s.	n.s.	***	n.s.	n.s.	n.s.
Chloroalkane and chloroalkene degradation	2.54E-01	1.96E-01	1.61E-01	1.70E-01	0.0110	n.s.	***	**	n.s.	n.s.	n.s.
Lipopolysaccharide biosynthesis	2.27E-02	4.52E-02	1.59E-03	1.53E-01	0.0113	n.s.	n.s.	n.s.	n.s.	n.s.	*
Pores ion channels	1.73E-01	1.94E-01	1.87E-01	3.10E-01	0.0119	n.s.	n.s.	***	n.s.	**	**
Cellular antigens	3.38E-02	1.62E-02	1.16E-02	1.71E-02	0.0122	n.s.	*	n.s.	n.s.	n.s.	n.s.
Translation proteins	8.42E-01	9.02E-01	9.47E-01	9.18E-01	0.0123	n.s.	***	n.s.	n.s.	n.s.	n.s.
Zeatin biosynthesis	3.74E-02	4.13E-02	4.58E-02	4.25E-02	0.0126	n.s.	**	n.s.	n.s.	n.s.	n.s.
Drug metabolism - other enzymes	3.10E-01	3.35E-01	3.82E-01	3.40E-01	0.0126	n.s.	**	n.s.	n.s.	n.s.	n.s.
Inorganic ion transport and metabolism	2.08E-01	1.72E-01	1.47E-01	2.41E-01	0.0128	n.s.	n.s.	n.s.	n.s.	n.s.	*
Amino acid metabolism	3.34E-01	2.86E-01	2.74E-01	2.39E-01	0.0128	n.s.	n.s.	***	n.s.	n.s.	n.s.
RNA transport	1.06E-01	1.39E-01	1.51E-01	1.63E-01	0.0131	n.s.	*	***	n.s.	n.s.	n.s.
Carbon fixation in photosynthetic organisms	5.14E-01	5.59E-01	5.51E-01	5.73E-01	0.0137	n.s.	n.s.	**	n.s.	n.s.	n.s.
Mismatch repair	7.33E-01	7.95E-01	8.59E-01	7.93E-01	0.0137	n.s.	***	n.s.	n.s.	n.s.	n.s.
Pyrimidine metabolism	1.68E+00	1.87E+00	2.04E+00	1.88E+00	0.0138	n.s.	***	n.s.	n.s.	n.s.	n.s.
Biosynthesis of vancomycin group antibiotics	3.71E-02	4.51E-02	4.62E-02	4.53E-02	0.0139	*	*	*	n.s.	n.s.	n.s.
Thiamine metabolism	4.51E-01	4.50E-01	4.93E-01	4.66E-01	0.0160	n.s.	n.s.	n.s.	n.s.	n.s.	n.s.
Other ion-coupled transporters	1.31E+00	1.12E+00	1.06E+00	1.18E+00	0.0171	n.s.	*	n.s.	n.s.	n.s.	n.s.
Photosynthesis	2.98E-01	3.28E-01	3.66E-01	3.34E-01	0.0184	n.s.	**	n.s.	n.s.	n.s.	n.s.
Shigellosis	8.84E-06	9.53E-07	0.00E+00	2.58E-05	0.0188	n.s.	n.s.	n.s.	n.s.	*	*
Electron transfer carriers	3.22E-02	1.64E-02	1.13E-02	3.91E-02	0.0200	n.s.	n.s.	n.s.	n.s.	*	*
General function prediction only	3.54E+00	3.62E+00	3.75E+00	3.64E+00	0.0203	n.s.	**	n.s.	n.s.	n.s.	n.s.
Bisphenol degradation	1.02E-01	5.41E-02	4.50E-02	4.79E-02	0.0204	*	*	*	n.s.	n.s.	n.s.
Polyketide sugar unit biosynthesis	1.32E-01	1.62E-01	1.73E-01	1.64E-01	0.0206	n.s.	n.s.	n.s.	n.s.	n.s.	n.s.
Peptidoglycan biosynthesis	7.78E-01	8.97E-01	9.83E-01	9.19E-01	0.0211	n.s.	*	n.s.	n.s.	n.s.	n.s.
Glycosyltransferases	2.51E-01	3.22E-01	3.65E-01	4.39E-01	0.0214	n.s.	n.s.	***	n.s.	n.s.	n.s.
Aminoacyl-tRNA biosynthesis	1.03E+00	1.12E+00	1.23E+00	1.13E+00	0.0215	n.s.	*	n.s.	n.s.	n.s.	n.s.
Terpenoid backbone biosynthesis	5.76E-01	5.54E-01	5.77E-01	5.14E-01	0.0219	n.s.	n.s.	**	n.s.	n.s.	***
Pentose phosphate pathway	7.66E-01	8.33E-01	8.22E-01	8.48E-01	0.0236	n.s.	n.s.	n.s.	n.s.	n.s.	n.s.
Novobiocin biosynthesis	1.74E-01	1.52E-01	1.43E-01	1.22E-01	0.0255	n.s.	n.s.	**	n.s.	n.s.	n.s.
Nitrogen metabolism	6.60E-01	6.23E-01	5.79E-01	6.61E-01	0.0264	n.s.	n.s.	n.s.	n.s.	n.s.	n.s.

Phosphatidylinositol signaling system	6.76E-02	8.71E-02	9.88E-02	1.12E-01	0.0269	n.s.	n.s.	*	n.s.	n.s.	n.s.
Vitamin B6 metabolism	1.43E-01	1.56E-01	1.66E-01	1.85E-01	0.0274	n.s.	n.s.	*	n.s.	n.s.	n.s.
Photosynthesis proteins	3.03E-01	3.30E-01	3.66E-01	3.40E-01	0.0276	n.s.	*	n.s.	n.s.	n.s.	n.s.
Protein export	5.04E-01	5.36E-01	5.76E-01	5.85E-01	0.0283	n.s.	n.s.	*	n.s.	n.s.	n.s.
Galactose metabolism	6.03E-01	8.64E-01	9.11E-01	8.91E-01	0.0287	n.s.	n.s.	n.s.	n.s.	n.s.	n.s.
Ion channels	1.14E-02	3.64E-02	4.91E-02	5.31E-02	0.0288	n.s.	n.s.	*	n.s.	n.s.	n.s.
Sphingolipid metabolism	6.65E-02	9.79E-02	1.06E-01	1.17E-01	0.0290	n.s.	n.s.	n.s.	n.s.	n.s.	n.s.
Protein kinases	2.77E-01	2.76E-01	2.64E-01	3.45E-01	0.0300	n.s.	n.s.	n.s.	n.s.	*	*
Sulfur metabolism	2.63E-01	2.57E-01	2.39E-01	2.79E-01	0.0317	n.s.	n.s.	n.s.	n.s.	n.s.	*
Isoflavonoid biosynthesis	7.43E-05	0.00E+00	0.00E+00	2.34E-05	0.0327	n.s.	n.s.	n.s.	n.s.	n.s.	n.s.
Protein folding and associated processing	5.35E-01	5.31E-01	5.23E-01	5.92E-01	0.0334	n.s.	n.s.	n.s.	n.s.	n.s.	n.s.
RNA polymerase	1.54E-01	1.81E-01	2.04E-01	1.86E-01	0.0335	n.s.	*	n.s.	n.s.	n.s.	n.s.
C5-Branched dibasic acid metabolism	2.53E-01	2.59E-01	2.56E-01	2.84E-01	0.0350	n.s.	n.s.	n.s.	n.s.	n.s.	n.s.
Biosynthesis of 12-, 14- and 16-membered macrolides	1.26E-05	4.24E-07	0.00E+00	6.56E-06	0.0351	n.s.	n.s.	n.s.	n.s.	n.s.	n.s.
Carbohydrate digestion and absorption	4.39E-03	1.74E-02	2.22E-02	2.76E-02	0.0370	n.s.	n.s.	*	n.s.	n.s.	n.s.
G protein-coupled receptors	0.00E+00	1.79E-05	1.56E-03	3.73E-04	0.0371	n.s.	n.s.	n.s.	n.s.	n.s.	n.s.
Pentose and glucuronate interconversions	4.75E-01	5.79E-01	5.70E-01	5.51E-01	0.0374	n.s.	n.s.	n.s.	n.s.	n.s.	n.s.
Primary immunodeficiency	3.87E-02	5.29E-02	6.02E-02	7.09E-02	0.0398	n.s.	n.s.	*	n.s.	n.s.	n.s.
Nucleotide excision repair	3.66E-01	3.58E-01	3.76E-01	3.34E-01	0.0399	n.s.	n.s.	n.s.	n.s.	n.s.	**
Isoquinoline alkaloid biosynthesis	8.23E-02	6.04E-02	4.88E-02	3.67E-02	0.0401	n.s.	n.s.	*	n.s.	n.s.	n.s.
Neuroactive ligand-receptor interaction	1.19E-06	0.00E+00	0.00E+00	0.00E+00	0.0420	*	***	****	n.s.	n.s.	n.s.
Pancreatic secretion	1.19E-06	0.00E+00	0.00E+00	0.00E+00	0.0420	n.s.	n.s.	n.s.	n.s.	n.s.	n.s.
Sesquiterpenoid biosynthesis	2.77E-06	0.00E+00	0.00E+00	0.00E+00	0.0420	*	n.s.	n.s.	n.s.	n.s.	n.s.
Various types of N-glycan biosynthesis	1.46E-06	0.00E+00	0.00E+00	0.00E+00	0.0420	*	n.s.	n.s.	n.s.	n.s.	n.s.
Biosynthesis and biodegradation of secondary metabolites	3.88E-02	2.62E-02	1.88E-02	5.11E-02	0.0423	n.s.	n.s.	n.s.	n.s.	*	**
Flagellar assembly	7.22E-01	6.11E-01	4.34E-01	3.76E-01	0.0441	n.s.	n.s.	n.s.	n.s.	n.s.	n.s.
Glycerolipid metabolism	3.95E-01	4.57E-01	4.67E-01	4.73E-01	0.0469	n.s.	n.s.	n.s.	n.s.	n.s.	n.s.
Amino acid related enzymes	1.35E+00	1.41E+00	1.49E+00	1.40E+00	0.0474	n.s.	n.s.	n.s.	n.s.	n.s.	n.s.
Pantothenate and CoA biosynthesis	5.54E-01	5.65E-01	5.94E-01	5.87E-01	0.0497	n.s.	n.s.	n.s.	n.s.	n.s.	n.s.

[a] Global comparison across the age groups based on Kruskal-Wallis test.

[b] Pairwise comparison across the age groups based on the *post hoc* Tukey-Kramer test.

Symbols: ****, $p < 0.001$; ***, $p < 0.01$; **, $p < 0.02$; *, $p < 0.05$.

Abbreviations: n.s., not significant; A/J, adults/juveniles; C(4–5M), cubs at age of 4–5 months; C(3M), cubs at age of 3 months; C(1–2M), cubs at age of 1–2 months.

Table S3. Statistical comparisons of fecal microbiota of the Eurasian otters by season [a].

Type	Diversity index	Statistical test	Global comparison	Pairwise comparison	
Alpha diversity	Chao1	Kruskal-Wallis test	n.s.	n.a.	n.a.
	Shannon	Kruskal-Wallis test	n.s.	n.a.	n.a.
Beta diversity	Jaccard similarity coefficient	PERMANOVA	*	Summer vs. Fall	*
				Summer vs. Winter	*
				Summer vs. Spring	n.s.
				Fall vs. Winter	n.s.
				Fall vs. Spring	n.s.
				Winter vs. Spring	n.s.
	Bray-Curtis similarity coefficient	PERMANOVA	*	Summer vs. Fall	*
				Summer vs. Winter	*
				Summer vs. Spring	n.s.
				Fall vs. Winter	n.s.
				Fall vs. Spring	n.s.
				Winter vs. Spring	n.s.

[a] The samples were grouped into spring (March, April, and May), summer (June, July, and August), fall (September, October, and November), or winter (December, January, and February) based on sampling dates.
Symbol: *, $p < 0.05$.
Abbreviations: n.s., not significant ($p > 0.05$); n.a., not applicable.
